# Intranasal Fentanyl in Preterm Infants Undergoing Peripherally Inserted Central Catheter Placement (INFENT PICC): A Feasibility Randomized Controlled Trial

**DOI:** 10.3390/children12091156

**Published:** 2025-08-30

**Authors:** Najla Tabbara, Shelley L. McLeod, Anna Taddio, Vibhuti Shah

**Affiliations:** 1 Department of Pharmacy, Mount Sinai Hospital, Toronto, ON M5G 1X5, Canada; 2 Institute of Health Policy, Management and Evaluation, Dalla Lana School of Public Health, University of Toronto, Toronto, ON M5T 3M6, Canada; 3 Schwartz/Reisman Emergency Medicine Institute, Sinai Health, Toronto, ON M5G 1X5, Canada; 4 Division of Emergency Medicine, Department of Family and Community Medicine, Temerty Faculty of Medicine, University of Toronto, Toronto, ON M5G 1V7, Canada; 5 Leslie Dan Faculty of Pharmacy, University of Toronto, Toronto, ON M5S 3M2, Canada; 6 Department of Paediatrics, Mount Sinai Hospital, Toronto, ON M5G 1X5, Canada

**Keywords:** fentanyl, intranasal drug administration, preterm infants, neonatal intensive care unit, peripherally inserted central catheter line insertion

## Abstract

**Background/Objectives**: Preterm infants in the neonatal intensive care unit (NICU) are subjected to clinically essential painful procedures including peripherally inserted central catheter (PICC) placement. Pharmacological interventions are inconsistently used for procedural analgesia due to concerns of adverse events. Intranasal (IN) fentanyl is a promising pharmacological alternative that delivers rapid targeted analgesia. The objectives of this blinded randomized controlled trial (RCT) were to assess the feasibility of conducting a definitive RCT of IN fentanyl for PICC placement in preterm infants and evaluate the acceptability and adoption of IN fentanyl for procedural pain management in the NICU. **Methods**: Infants admitted to the NICU (Mount Sinai Hospital, Toronto) with a gestational age (GA) at birth < 32 weeks undergoing their first PICC placement were randomized to IN fentanyl or placebo as an add-on to standard of care. The primary feasibility outcomes were recruitment and completeness of data collection for pain assessment. The pre-specified targets were recruitment of 24 participants and at least 80% of video-recordings being suitable for pain assessment. Secondary outcomes were adverse events, and IN fentanyl acceptability and adoption by healthcare providers. (ClinicalTrials.gov ID NCT06590870). **Results**: Between August 2024 and January 2025, 16 parents/guardians were approached resulting in eight enrollments for a consent rate of 50% (95% CI 28, 72). The target recruitment of 24 participants was not achieved. Out of six infants that received study interventions, all video-recordings were suitable for pain assessment by outcome assessors with a success rate of 100% (95% CI 61, 100). There were no adverse events. Fifteen healthcare providers completed the survey and reported acceptability of IN fentanyl but barriers with its adoption in clinical practice. **Conclusions**: Major modifications to the recruitment strategy would be required to progress to a definitive RCT. Strategies facilitating IN fentanyl adoption for procedural analgesia in the NICU are needed.

## 1. Introduction

As part of their clinical management, infants admitted to the neonatal intensive care unit (NICU) are exposed to multiple painful procedures with an average of 7.5 to 17.3 invasive procedures per day during the first two weeks of life or admission to hospital [1]. In very preterm infants (gestational age [GA] at birth < 32 weeks), early-life and repeated exposure to pain between birth and a postmenstrual age of 32 weeks is associated with short- and long-term negative sequela, including alterations in pain responsivity, changes in motor and cognitive functioning, reduced grey and white matter, and thalamic volume loss [2,3,4]. Despite these deleterious effects, pain management strategies continue to be underutilized in NICUs worldwide [5,6,7,8].

Placement of a peripherally inserted central catheter (PICC) is a common clinically essential painful procedure performed in very preterm infants [9]. Several phases of the PICC placement could potentially contribute to pain including skin preparation prior to the procedure, application of a tourniquet for vessel distension, needle insertion, catheter insertion and threading, and securement of the PICC [10]. Assessments during PICC placement indicate the procedure elicits moderate to severe pain responses [11,12]. Strategies to minimize and manage infant procedural pain consist of non-pharmacological and pharmacological interventions. Non-pharmacological interventions such as non-nutritive sucking and sweet-tasting solutions (e.g., sucrose) are the mainstay of analgesic therapy [13,14,15,16,17]. For moderately to severely painful procedures such as PICC placement, non-pharmacological interventions alone have variable effectiveness and multi-modal analgesia is favoured [13,16,17,18]. Pharmacological alternatives that can be combined with non-pharmacological strategies include topical anesthetics, non-opioid analgesics, and opioids [19,20,21,22,23,24,25]. Topical anesthetics have not been shown to reduce pain during PICC placement, and their application can contribute to local skin reactions (redness, swelling, blanching) and carries the risk of methemoglobinemia [19,20,21,22,23]. In a single randomized controlled trial (RCT) comparing three doses of intravenous acetaminophen, there was no difference in acute pain during PICC placement [24]. In terms of opioids, pain reduction was demonstrated in one RCT evaluating intravenous morphine infusion and a second RCT studying intravenous remifentanil infusion for PICC placement [21,25].

Opioids are however inconsistently used in clinical practice due to concerns of adverse events, specifically cardiovascular instability (e.g., hypotension) and cortical neuronal activity depression, resulting in suboptimal pain management [5,6,7,8,26]. Intranasal (IN) analgosedative agents have emerged as attractive alternatives in infants as they provide rapid targeted analgesia and allow drug absorption with minimal discomfort [27]. Two Canadian observational studies evaluated the effectiveness of IN fentanyl for painful procedures in the NICU, including PICC placements, and found that IN fentanyl can reduce procedural-related pain with a favourable safety profile [28,29]. Experience with IN fentanyl use has also been reported for other painful procedures, including three RCTs for retinopathy of prematurity screening [30,31,32,33,34,35].

Randomized controlled trials are considered the gold standard to assess the efficacy and safety of new interventions. Feasibility studies are important in planning full-scale definitive RCTs and determining whether they can or should be pursued by evaluating uncertainties with feasibility and identifying potential problems with participant recruitment and retention, randomization, blinding, study interventions, data collection procedures, and trial management [35,36,37]. Key areas of uncertainty in neonatal RCTs are recruitment, which can be challenging due to infant vulnerability and parental stress, and outcome ascertainment [38,39,40,41]. When proposing a new intervention into clinical practice, studying implementation outcomes can also bridge the gap between evidence and practice, and identify opportunities for effective implementation strategies. Acceptability captures the perception among implementation stakeholders that a new treatment is satisfactory while adoption aims to understand uptake of an intervention [42]. The primary objective of the IN fentanyl in preterm infants undergoing PICC placement (INFENT PICC) feasibility RCT was to address uncertainties with participant recruitment and completeness of data collection. The secondary objective was to survey NICU healthcare providers regarding their acceptability and adoption of IN fentanyl in clinical practice.

## 2. Materials and Methods

### 2.1. Study Design and Setting

This was a single centre randomized, blinded, parallel group, feasibility trial conducted at the academic level III NICU at Mount Sinai Hospital, Toronto, ON, Canada between August 2024 and January 2025. Randomization and preparation of study interventions were performed by the clinical trials pharmacy. Infants and their parents/guardians, the healthcare team, study investigators, and outcome assessors were blinded to the randomization schedule and interventions received during the trial. While the NICU operated continuously (24/7 unit), the clinical trial operating hours were Monday to Friday from 07:00 to 19:00, except for holidays, for completion of study procedures owing to the pharmacy’s schedule to dispense study medications.

### 2.2. Participants

Infants with a GA at birth < 32 weeks and/or birth weight < 1.5 kg undergoing their first PICC placement and considered medically appropriate for the study by the most responsible physician were eligible for inclusion. Exclusion criteria were infants with choanal atresia, nasal mucosal erosion, epistaxis, facial anomalies, genetic conditions known to affect neurological development, severe intraventricular hemorrhage, cardiopulmonary instability managed with inotropes, vasopressors, phosphodiesterase enzyme inhibitors, neuromuscular blocking agents at the time of PICC placement, bronchopulmonary dysplasia, concomitant continuous intravenous infusions or scheduled enteral doses of opioids or sedatives within 12 h of PICC placement, concomitant strong CYP3A4 inhibitors at the time of PICC placement, or a previous documented adverse reaction to any formulation of fentanyl. A member of the research team who was not involved in the infant’s clinical care obtained informed consent from parents/guardians.

### 2.3. Randomization

Infants were randomly assigned to either IN fentanyl or placebo with a 1:1 allocation ratio using a computerized random number generator (unrestricted randomization). In the case of multiple births (e.g., twins), eligible infants were planned to be randomized independently of each other. Participants were randomized using an online central randomization service by the clinical trials pharmacy before the planned PICC placement, with no insight into the sequence generation. Assignments were only known to the necessary clinical trials pharmacy personnel for preparation and dispensing of study interventions with appropriate concealed labelling. The randomization code was maintained by the clinical trials pharmacy and only released at the end of the study.

### 2.4. Interventions

#### 2.4.1. Standard of Care

The standard of care at Mount Sinai Hospital for PICC placement is oral sucrose 24% solution applied to the anterior tip of the tongue two minutes before needle insertion (the skin-breaking component of the procedure). Sucrose dosing is based on current infant weight as follows: weight < 1.5 kg, 0.2 mL of sucrose; weight 1.5–2.5 kg, 0.5 mL of sucrose; and weight > 2.5 kg, 1 mL of sucrose as per the institutional protocol. Sucrose administration was followed with a pacifier as a form of non-nutritive sucking if participant status allowed.

#### 2.4.2. IN Fentanyl or Placebo

The fentanyl solution was prepared by diluting 2 mL of fentanyl 50 µg/mL (by SteriMax Inc., Oakville, ON, Canada) with 8 mL of bacteriostatic 0.9% sodium chloride (by Pfizer Inc., Kirkland, QC, Canada) for a final 10 µg/mL fentanyl solution. The placebo was bacteriostatic 0.9% sodium chloride and did not require further dilution. Participant specific doses were prepared and dispensed by the clinical trials pharmacy for immediate usage before the PICC placement. A nurse trained in IN medication delivery administered one dose of fentanyl 1.5 µg/kg (or equivalent volume placebo) in one nostril using a mucosal atomization device (MED-RX^®^ Nasal Atomizer converting solutions into a fine mist of 30–100 µm particles, supplied by Canadian Hospital Specialties Ltd., Oakville, ON, Canada) ten minutes before needle insertion. In children with moderate to severe acute pain, IN fentanyl at an established mean dose of 1.5 µg/kg/dose provides analgesia without any serious adverse events (e.g., chest wall rigidity/wooden chest syndrome, hemodynamic instability, respiratory compromise) [43,44]. An additional 0.1 mL of solution was added to the volume for administration to account for mucosal atomization device dead space. At the NICU at Mount Sinai Hospital, all nurses had received IN medication administration training as part of their education requirements.

#### 2.4.3. Co-Interventions

Non-pharmacological strategies were permitted (e.g., swaddling). Noise and light illumination were not altered from standard clinical practice. Participants prescribed other analgesics for non-procedural analgesia (e.g., ibuprofen for patent ductus arteriosus treatment) at the time of PICC placement continued these medications.

### 2.5. Outcomes

#### 2.5.1. Recruitment

All infants with a GA at birth < 32 weeks and/or birth weight < 1.5 kg undergoing PICC placement were screened daily for study enrollment. The screening and enrollment log captured the date of screening, the number of infants assessed for eligibility, the number of infants approached for consent, the number of enrolled infants, and the number of participants completing study procedures. Reasons for ineligibility, inability to be enrolled, or not completing study procedures were also recorded. The recruitment rate was the number of enrolled infants per month, and the consent rate was the proportion of eligible infants who consented among those approached. For parents/guardians that declined participation in the trial, reasons for non-participation were collected if provided.

#### 2.5.2. Completeness of Data Collection for Pain Score Assessment

Assessment of pain scores was completed after the procedures by two trained and blinded outcome assessors using the premature infant pain profile-revised (PIPP-R) tool. Infants’ faces were video recorded for the entire duration of the procedure using a digital camera (FDR-AX43A/B AX43A 4K Handycam^®^ with Exmor R™ CMOS sensor, supplied by Sony Electronics Inc., San Diego, CA, USA). Physiological indicators of pain were also captured using a second digital camera from the standard cardiopulmonary monitor in each NICU room. All video-recordings were completed by a member of the research team, and video-recording procedures were piloted and standardized in a simulation environment before any participant enrollment. Based on the scoring instructions for PIPP-R, for each phase of the procedure, infants were assessed for 15 s at rest and for 30 s after the start of each phase to calculate a PIPP-R score. Scores range from 0 to 21 and are interpreted as no pain (score of 0), mild pain (score 1–6), moderate pain (score 7–12), or severe pain (score 13–21) [45,46]. Observer agreement was measured using PIPP-R scores from the two outcome assessors.

The pre-specified criteria for feasibility and progression to a definitive RCT were:

- A consent rate of 50% and recruitment of four infants per month over a 6-month period: in 2022, there was an average of 10 PICC placements per month in infants with a GA at birth < 32 weeks at Mount Sinai Hospital based on data captured in the Canadian Neonatal Network database (personal communication). Assuming eight infants meet eligibility criteria and a 50% consent rate, the target of four infants per month was derived.
- At least 80% of video-recordings suitable for pain score assessment at the needle insertion phase of the PICC placement by blinded outcome assessors: this target was chosen as equipment failures preventing pain score assessment have been reported to be as high as 20% in previous RCTs of procedural analgesia for PICC placement [21,22,23,24,47].

#### 2.5.3. Adverse Events

The following adverse events were abstracted from the NICU cardio-respiratory safety monitoring system and the electronic medical record: number of apneas (cessation of breathing > 20 s), number of bradycardias (heart rate < 100 beats per minute), number of desaturations (oxygen saturation < 80%), chest wall rigidity, escalation in ventilatory support (change in mode, settings, or fraction of inspired oxygen), and need for other analgesia (name of medication, dose, administration route, frequency, and time of prescribing). Adverse events were collected in one hour time intervals post-IN intervention administration. Based on the pharmacokinetic profile of IN fentanyl and clustered care in the NICU, the observation period was 6 h after IN fentanyl or placebo administration.

#### 2.5.4. Acceptability and Adoption of IN Fentanyl

All NICU healthcare providers involved with study procedures were invited to complete a standardized 9-item paper-based questionnaire immediately after the PICC placement. Using a Likert scale, survey respondents ranked the acceptability of IN medication administration, specifically satisfaction with its administration and effect on the infant. For adoption, barriers and enablers to IN fentanyl use in preterm infants were captured using multiple answers from a defined list of choices or open-ended responses. Prior to distribution, the questionnaire was peer-reviewed by three NICU healthcare professionals, who were not part of the survey design and not candidates for participation in the survey, for testing of face validity, clarity and interpretation of the questions, and ease of administration.

### 2.6. Sample Size

The sample size was based on the primary feasibility outcomes. The target sample size for this RCT was 24 (12 infants per group) and permitted up to 30 (15 infants per group). Study investigators justified these numbers to be sufficient to inform participant recruitment as well as adequate to establish feasibility of data collection procedures [48,49].

### 2.7. Blinding

This was a blinded RCT: participants (infants with their parents/guardians), care providers (NICU healthcare team), investigators, and outcome assessors were blinded. The clinical trials pharmacy maintained the randomization code and the inventory of study interventions. Both fentanyl and placebo are clear solutions, indistinguishable by colour, smell, or viscosity. They were dispensed in identical syringes with an attached mucosal atomized device and stored in the NICU’s narcotic and controlled substance cupboard until ready for administration. There was no emergency unblinding as the intervention was administered only once pre-procedure.

### 2.8. Analytic Methods

This RCT was designed to test study procedures and calculate the proportion of infants who meet feasibility objectives. For the feasibility objective of consent rate, it was calculated as the number of enrolled infants divided by the number of infants approached for consent. For the feasibility objective of completion of data collection for pain assessment, only infants administered study interventions and completing the PICC placement were assessed. Completeness of data collection for pain assessment at the needle insertion phase of the PICC placement was calculated as the number of infants with suitable video-recordings (assessed by blinded outcomes assessors) divided by the number of infants completing study procedures. To calculate the 95% confidence intervals (CI) for the estimates of consent rate and success rate of suitable video-recordings, the Wilson method was used due to the small sample size [50].

Participant demographic and clinical characteristics, PIPP-R scores, and adverse events were summarized using measures of central tendency: median and interquartile range (IQR) for continuous variables, and count and proportion for categorical variables. Between-group comparisons were not performed as this RCT was not powered for these analyses and did not aim to draw inferences. To assess the degree of agreement between outcome assessors for pain assessments, the intraclass correlation coefficient (ICC) was calculated using a two-way random effects model and interpreted as follows: <0.5 poor reliability, 0.5–0.74 moderate reliability, 0.75–0.9 good reliability, and >0.9 excellent reliability [51]. Descriptive analyses were performed using R Studio version 4.4.2 (Vienna, Austria) [52]. Survey data were summarized using count and proportion for categorical variables and themes for free-text responses.

## 3. Results

### 3.1. Study Infants

**Figure 1:**
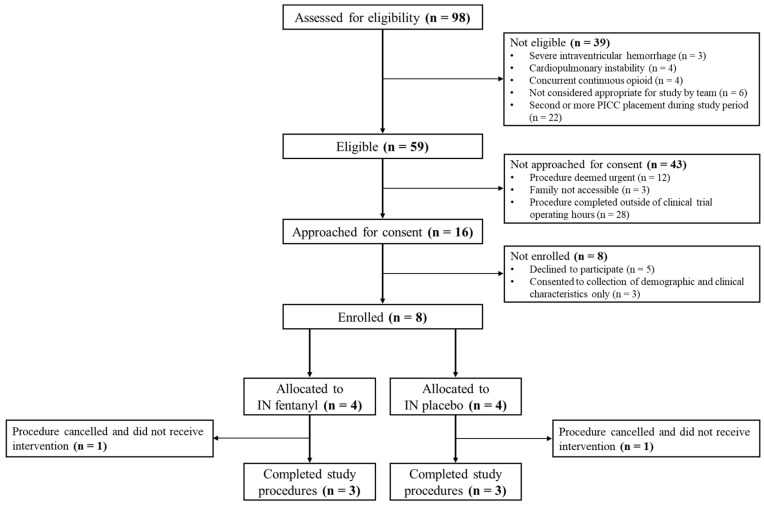
Participant flow diagram.

A participant flow diagram is presented (Figure 1). Over the 6-month study period, 98 infants were assessed for eligibility. Thirty-nine (40%) infants were not eligible: eleven had an exclusion criterion affecting response to fentanyl or pain assessment (severe intraventricular hemorrhage, cardiopulmonary instability, or concomitant opioid administration), six were not considered appropriate for study enrolment by the primary healthcare team, and 22 had more than one PICC placement during the study period. Among the six infants not considered appropriate for the study, the healthcare team provided compassionate reasons for non-participation including a deterioration in clinical status (n = 2), new diagnosis requiring transfer to a level IV NICU (n = 1), or approach by another NICU clinical study the same day of INFENT PICC’s planned approach (n = 3).

Of the 59 infants eligible for the study, the research team was unable to approach 43 (73%) parents/guardians due to urgent PICC placement for umbilical venous catheter complications or loss of intravenous access (n = 12), families not being accessible to provide consent (n = 3), or procedures being completed outside of clinical trial operating hours (n = 28). Out of 59 eligible procedures, 16 (27%) were completed on weekends, 8 (14%) on weekdays during the overnight shift (between 19:00 and 07:00), and four (7%) during a holiday. Sixteen parents/guardians were approached for their infant’s enrolment in the trial. Eight infants were enrolled and six received the intended treatment, as two procedures were cancelled due to a change in clinical status within one hour of the planned PICC placements. The demographic and clinical characteristics of infants are displayed (Table 1).

### 3.2. Feasibility Outcomes

Out of 16 infants, the consent rate was 50% (95% CI 28, 72). Reasons provided by parents/guardians for non-participation were at least one of the following: concerns with potential adverse events of fentanyl (including its reputation as an illicit drug and association with addiction), extremely low birth weight infant (<1 kg), or disinterest in clinical research. The target recruitment of 4 infants per month was not achieved.

### 3.3. Pain Assessment

The success rate of suitable video-recordings for pain assessment at the needle insertion phase was 100% (95% CI 61, 100) in the six infants completing study procedures. For assessment of observer agreement, PIPP-R scores provided by each outcome assessor for all PICC placement phases were used to calculate the ICC. Pain scores are presented in Table 2. Agreement between outcome assessors for pain assessments was 0.76 (95% CI 0.57, 0.87) suggesting good reliability.

### 3.4. Healthcare Provider Survey

Sixteen healthcare team members were eligible to complete the survey. The response rate was 94% (15/16). Their roles in the INFENT PICC study were as follows: five (33%) PICC inserters and 10 (67%) procedure assistants. Most respondents (n = 11; 73%) were registered nurses in addition to three (20%) physicians. Six (48%) respondents administered the IN study intervention with high intervention fidelity as there were no issues encountered with administration. Survey responses are summarized in Table 3.

To assess acceptability with IN medications, healthcare providers were asked to rate their experience with giving or observing the administration of the intervention and how they viewed its impact on the infant. To the statement “giving/observing the IN medication was stressful for me”, most respondents strongly disagreed (n = 11; 73%) or disagreed (n = 2; 13%) and only two respondents agreed (n = 1; 7%) or strongly agreed (n = 1; 7%) with the statement. To the statement “receiving the IN medication was stressful for the baby”, most respondents also strongly disagreed (n = 8; 53%) or disagreed (n = 6; 40%) and one (7%) healthcare provider had a neutral stance; no healthcare providers agreed or strongly agreed with the statement.

In exploring adoption of IN fentanyl in clinical practice, six (40%) respondents reported a lack of comfort with the IN administration route, three (20%) had concerns with the efficacy of IN fentanyl, and one (7%) had concerns about adverse events. From open-ended responses, two (13%) healthcare providers stated that they were not confident with the preparation of the IN fentanyl solution in practice (dilution and attachment of the mucosal atomization device). Among those that administered the IN medications, they reported that it was either their first or second time only administering medications via the IN route in a clinical setting. In terms of enablers, the institutional IN fentanyl guideline was endorsed by 13 (87%) respondents, seven (47%) would welcome additional education and training with IN administration, and 11 (73%) wanted increased clinical experience to feel more comfortable with IN fentanyl use in preterm infants. While blinded to the allocation to IN fentanyl or placebo, four (27%) healthcare providers described in free-text responses that the infant was comfortable during the procedure after administration of the IN intervention and that the PICC placement went smoothly, underlying a theme of potential for improved care.

## 4. Discussion

Based on a priori feasibility and progression criteria, the INFENT PICC feasibility RCT did not recruit to the target sample size of 24 participants in 6 months despite a consent rate of 50% among approached parents/guardians. Nonetheless, there was 100% success in video-recordings for pain assessment at the needle insertion phase of the PICC placement which would serve as the primary outcome in a future definitive RCT. The major challenge of the study was a high screen failure rate of 73% where eligible infants could not be approached for consent or enrolled in the trial. This was largely attributed to PICC placements completed outside of clinical trial operating hours when the clinical trials pharmacy could not perform the randomization and dispense study interventions. For urgent PICC placements or when parents/guardians could not be present in the NICU at the time of planned study approach, these scenarios cannot be overcome with amendments to recruitment in a future definitive RCT without increasing the burden on families (e.g., calling parents/guardians when their infant requires an unplanned urgent procedure). While the clinical trials pharmacy ensured appropriate randomization, allocation concealment, intervention preparation, and blinding, there was a trade-off between the study’s internal validity and recruitment target. Continuing with the pharmacy generating the randomization and allocation sequence prior to trial commencement, a strategy to optimize recruitment is the preparation of sequentially numbered opaque envelopes and intervention containers to be stored in the NICU’s narcotic and controlled substance cupboard. This would allow for the approaching of parents/guardians on evenings, weekends, and holidays. However, a healthcare professional/research team member would need to dilute study interventions to the correct concentration, dispense them with concealed labelling, and ensure no compromise to blinding procedures. With 24/7 dedicated and trained research staff and sufficient funding, this limitation may be addressed in a full-scale definitive RCT. Alternatives approaches supporting informed consent also include antenatal consent or a two-stage consent (verbal consent followed by written consent) which can be explored in certain jurisdictions and in consultation with research ethics boards. Further feasibility assessments of amended recruitment strategies are warranted.

In terms of an issue that is likely to be a problem in both the trial and real-world setting, there is a need to involve parents in study design and conduct in order to optimize the recruitment strategy and capture patient-relevant outcomes, as well as understand obstacles with their acceptability of IN fentanyl. All pharmacological interventions are associated with side effects and IN fentanyl’s side effect profile currently consists of apneas and desaturations (which were not detected in this small study). In consent discussions, it would be of value to underline that apneas, bradycardias, and desaturations are common in preterm infants at baseline, even without the administration of medications. A parent-partner would be an excellent research team member to contribute parents’ lived experiences in the NICU and their perspective on participating in clinical research. In addition, parenteral involvement during and after PICC placement procedures should be explored further. Parents have reported that infant pain and its inadequate management is distressing, and strategies to empower parents and actively engage them in pain management (e.g., parental presence or voice) may be of benefit [53].

Acceptability and adoption of IN fentanyl by the healthcare team is an important issue in clinical practice. In the survey, IN medication administration was acceptable to most NICU healthcare providers. They were able to complete administrations successfully, despite limited previous experience with IN fentanyl administration. Furthermore, healthcare providers were satisfied that IN interventions did not appear to add to infant pain or distress. As a result, they stated that participating in the INFENT PICC study increased their confidence for future IN medication administrations. However, for the adoption of IN fentanyl for procedural analgesia, opportunities were identified to provide additional training and education on IN administration. Healthcare providers wished to have additional experience with its use in preterm infants. Interestingly, uncertainty with the efficacy of IN fentanyl was a barrier reported by more respondents than concerns with its potential adverse events.

The survey results emphasize the importance of assessing the suitability of a new intervention from the perspective of healthcare providers, in addition to determining the feasibility of conducting the RCT to determine intervention efficacy and safety [54]. For procedural pain in the NICU, acceptability of both non-pharmacological and pharmacological pain management strategies is not well documented, and adoption continues to be suboptimal for many painful procedures resulting in undertreated pain [5,6,7,8]. One strategy to improve the adoption of pain management interventions was highlighted in the study of the Implementation of Infant Pain Practice change resource which was developed by a multidisciplinary Canadian research team. This constitutes an eHealth tool to enhance procedural pain assessment and the use of pain management interventions. In an effectiveness-implementation study in 23 NICUs across Canada, use of this resource increased the number of pain assessments as well as the provision of non-pharmacological pain management strategies, compared to pre-implementation data [55]. A similar platform and study focusing on pharmacological pain management interventions could be of benefit to NICU clinical providers. Pertaining to knowledge enhancement and comfort/practice with IN medication administration, didactic education sessions combined with simulation-based training should be pursued [56,57].

A limitation of the survey was the representativeness of respondents. Due to the small sample size of the INFENT PICC study, a limited number of healthcare providers participating in study procedures were eligible to complete this survey. Potential sampling bias would impact the generalizability of survey results to all NICU healthcare professionals. In terms of survey design, there is an opportunity to complete further validity (content and construct) and reliability assessments to improve the performance of the questionnaire.

## 5. Conclusions

With the expansion of study recruitment hours, family engagement and involvement, and modifications to the intervention allocation and preparation, a full-scale definitive RCT evaluating the impact of IN fentanyl on procedural pain management for PICC placement could be feasible. Intranasal medication administration was acceptable to NICU healthcare providers and did not interfere with care delivery. Strategies are necessary to enhance adoption of IN fentanyl and reduce barriers to its implementation in clinical practice.

## Figures and Tables

**Table 1:** INFENT PICC participant characteristics.

Characteristic	Fentanyl Group (n = 4)	Control Group (n = 4)
Gestational age at birth, weeks	25.5 (24.7, 26.3)	23.4 (23, 25.3)
Birth weight, kg	0.79 (0.69, 0.96)	0.64 (0.52, 0.82)
Female sex	1 (25)	2 (50)
Apgar score (1 min)	7 (5, 8)	2 (2, 4)
Apgar score (5 min)	9 (8, 9)	9 (8, 9)
Postnatal age, days	6 (5, 6)	5 (4, 6)
Postmenstrual age, weeks	26.2 (25.5, 27)	23.9 (23.6, 25.8)
Weight at PICC placement, kg	0.79 (0.63, 0.98)	0.55 (0.52, 0.7)
Invasive ventilatory support	2 (50)	3 (75)

Presented as median (IQR) or count (percentage).

**Table 2:** Pain assessment at PICC placement phases.

PIPP-R Score by PICC Placement Phase	Fentanyl Group (n = 3)	Control Group (n = 3)
Intranasal solution administration	3 (1.5, 5)	9 (4.5, 10)
Oral sucrose administration	3 (1.5, 3)	4 (2, 6) *
Skin preparation	0 (0, 3.5)	7 (3.5, 10)
Needle insertion	0 (0, 1.5)	9 (7.5, 11.5)
Second needle insertion	3 ^†^	8.5 (8, 9) ^‡^
Catheter insertion and threading	0 (0, 1.5)	9 (4.5, 9.5)
Recovery	0 (0, 0)	3 (1.5, 3)

Presented as median (IQR); * one infant in the control group was not administered sucrose; † one infant in the fentanyl group required a second venipuncture; ‡ two infants in the control group required a second venipuncture.

**Table 3:** Healthcare provider survey results.

Questions	Responses (n = 15)
**Giving/observing the IN medication was stressful for me**	
Strongly disagree	11 (73%)
Disagree	2 (13%)
Neither agree nor disagree	0
Agree	1 (7%)
Strongly agree	1 (7%)
**Receiving the IN medication was stressful for the baby**	
Strongly disagree	8 (53%)
Disagree	6 (40%)
Neither agree nor disagree	1 (7%)
Agree	0
Strongly agree	0
**What are your barriers to IN fentanyl use in preterm infants? Check all that apply**	
Lack of comfort with IN administration route	6 (40%)
Concerns about efficacy	3 (20%)
Concerns about adverse events	1 (7%)
**What are your enablers to IN fentanyl use in preterm infants? Check all that apply**	
Institution-specific IN fentanyl guideline	13 (87%)
Additional education and training on IN administration	7 (47%)
Clinical experience with IN administration	11 (73%)

## Data Availability

The data presented in this article are not readily available because of privacy and ethical reasons. Requests to access the data should be directed to the corresponding author.

## Abbreviations

The following abbreviations are used in this manuscript:

CI	Confidence interval
GA	Gestational age
ICC	Intraclass correlation coefficient
IQR	Interquartile range
IN	Intranasal
NICU	Neonatal intensive care unit
PICC	Peripherally inserted central catheter
PIPP-R	Premature infant pain profile-revised
RCT	Randomized controlled trial
